# Differential Impact of Interference on Internally- and Externally-Directed Attention

**DOI:** 10.1038/s41598-018-20498-8

**Published:** 2018-02-06

**Authors:** David A. Ziegler, Jacqueline R. Janowich, Adam Gazzaley

**Affiliations:** 1 Department of Neurology, University of California San Francisco, San Francisco, California USA; 2 Neuroscape, University of California San Francisco, San Francisco, California USA; 3 Department of Psychology, University of New Mexico, Albuquerque, New Mexico USA; 4 Departments of Psychiatry and Physiology, University of California San Francisco, San Francisco, California USA

## Abstract

Attention can be oriented externally to the environment or internally to the mind, and can be derailed by interference from irrelevant information originating from either external or internal sources. However, few studies have explored the nature and underlying mechanisms of the interaction between different attentional orientations and different sources of interference. We investigated how externally- and internally-directed attention was impacted by external distraction, how this modulated internal distraction, and whether these interactions were affected by healthy aging. Healthy younger and older adults performed both an externally-oriented visual detection task and an internally-oriented mental rotation task, performed with and without auditory sound delivered through headphones. We found that the addition of auditory sound induced a significant decrease in task performance in both younger and older adults on the visual discrimination task, and this was accompanied by a shift in the type of distractions reported (from internal to external). On the internally-oriented task, auditory sound only affected performance in older adults. These results suggest that the impact of external distractions differentially impacts performance on tasks with internal, as opposed to external, attentional orientations. Further, internal distractibility is affected by the presence of external sound and increased suppression of internal distraction.

## Introduction

Our ability to manage the barrage of sensory inputs that we encounter in the world is what allows us to make appropriate decisions and engage in complex, goal-directed behavior^1^. Allocation of attentional resources is essential for such goal-directed behaviors, and attention can be divided into external and internal subsystems^2,3^. Behaviors that are “externally-oriented” are those that depend on the presence of external stimuli (e.g., paying attention to external visual or auditory stimuli, reading, or memory encoding); behaviors that are internally-oriented are those that occur in the absence of any external stimuli (e.g., planning, memory retrieval, calculation, interoception/awareness of internal states and mental imagery). An obstacle to achieving high-level performance on attention-demanding tasks is interference by both external and internal distraction. Just as goal-directed activities can be derailed by interference from irrelevant stimuli in the external environment, interference can also arise from the internal milieu in the form of intrusive thoughts, emotions, and urges^4–8^, or as a complex interaction between these two sources.

### Impact of External Interference

The vast majority of studies have focused on the impact of external interference on performance of a primary task, likely due to the convenience of being able to manipulate external factors in a laboratory environment. Externally-presented visual and auditory distractions have been shown to impair performance on numerous externally-oriented tasks^9^, including episodic memory retrieval^10–12^, categorization performance^13^, and attention^14,15^, and also on internally-oriented tasks such as working memory^16,17^. Further, we have shown that early engagement of top-down control mechanisms can impact stimulus processing and minimize distraction costs associated with external interference^17,18^. Further, resistance to the negative impact of distraction on working memory involves maintaining functional connectivity between the prefrontal cortex (PFC) and visual cortical regions^19–21^.

Studies of cognitive aging have shown that healthy older adults (OA) are particularly susceptible to interference, likely contributing to deficits in cognitive control and attention regulation^22,23^. Specifically, OA show impairments in both focusing and distributing attention in space^24^, time^25^, and toward important object features^26–29^. Such failures of attentional regulation likely contribute to an array of age-related cognitive impairments. Of particular note, we have shown that OA experience deficits in the suppression of externally presented distracting information that in turn has a negative impact on task performance^18,23,30–36^.

### Impact of Internal Interference

Prior studies of internal interference have focused on what is referred to as internal distractions or intrusions, which have been conceptualized as ‘mind-wandering’^8,37^, ‘stimulus-independent thought’^38^, ‘self-generated thought’^39^, ‘task-unrelated thought’^40^, ‘spontaneous cognition’^41^, ‘introspectively oriented thought’^40^, and more indirectly as ‘spontaneous fluctuations of attention’^42^. Semantic differences aside, the fundamental characteristic of internal interference is that attention is derailed from an original task or goal and instead becomes focused on internal thoughts. This shift in attentional focus may be either intentional (i.e., diversions) or unintentional (i.e., intrusions) and can occur with or without awareness. Studies of the frequency of ‘mind-wandering’ show that between 30–50% of our waking thoughts are ‘stimulus-independent’ or not related to the primary task or goal at hand^43–45^. These off-task thoughts occur during almost every type of behavior and task that has been monitored^43,44^, and they result in demonstrable costs in task performance^46^.

Given the well-documented age-related declines in cognitive control and in external distractor suppression^33,47,48^, one might predict an increased susceptibility of OA to internal distractions. Surprisingly, several studies have shown that OA report significantly fewer internal distractions on a variety of tasks^49–51^. One interpretation of these findings is that OA have insufficient resources to maintain both task-relevant and irrelevant thoughts; this notion is consistent with the view that mind-wandering results from failures of cognitive control.

### Interactions between Internal and External Attention and Interference

Surprisingly, relatively few studies have explored the nature and underlying mechanisms of interference in the context of differing attentional orientation (i.e., internally-focused or externally-oriented). Studies from our lab have shown that external distraction interacts with both externally-oriented tasks, such as memory encoding and attention^52,53^; as well as internally-oriented tasks, such as memory recall^10,11^. A few studies have attempted to link internal distractions to externally-directed attention by examining correlations between the propensity for mind-wandering and performance on attention demanding tasks^54^. To our knowledge, however, no studies have evaluated how attention orientation interacts with both external distraction and the regulation of internal distractions in a common experimental framework. For example, it is unknown whether it is more difficult to regulate internal distracting thoughts when one is engaged in an internal monitoring task vs. an external monitoring task and how this is modulated by external noise. The goal of this experiment was to study internal and external distraction during both internally- and externally-oriented tasks to understand how different attentional orientations impact distraction regulation and whether these processes change during the course of healthy aging.

## Materials and Methods

### Participants

The participants for the study were 25 healthy younger adults (YA) between the ages of 18 and 30 years (mean = 23.1 +/− 2.1; 13F, 12M) and 25 healthy older adults (OA) between the ages of 60 and 72 years (mean = 64.5 +/− 5.3; 14F, 11M). Data were excluded for 1 YA and 2 OA due to technical or procedural problems (it was determined after the end of the experiment that one OA had removed the headphones for half of the trials and the headphones malfunctioned for the other two excluded participants). All participants were fluent English speakers and had normal or corrected-to-normal vision (screened using a Snellen chart). Our exclusion criteria were: history of neurological or psychiatric disease, use of psychoactive medications, substance misuse, and presence of serious medical conditions, including history of heart disease, diabetes, and untreated hypertension. Those participants whose hypertension was controlled by prescription medication were admitted into the study. All participants gave informed consent and received monetary compensation for their participation. All methods were carried out in accordance with relevant guidelines and regulations approved by the UCSF Committee on Human Research’s Institutional Review Board.

To be included in this experiment, we required that all older adults be deemed cognitively normal (within 1.5 *SD*s) relative to normative values for age-matched controls based on a neuropsychological assessment that occurred within two years of being recruited to this study. The neuropsychological evaluation included the following tests: Mini Mental State Exam (MMSE), geriatric depression scale (GDS), California Verbal Learning Test—Second Edition (CVLT-II) working memory and verbal learning, Wechsler Adult Intelligence Scale-Revised (WAIS-R) incidental recall, Stroop interference test, and the Trail-Making Test (A and B) of visual-motor sequencing, semantic fluency, and phonemic fluency measures from the Delis–Kaplan Executive Function System (D-KEFS).

### Stimuli and cognitive tasks

**Figure 1:**
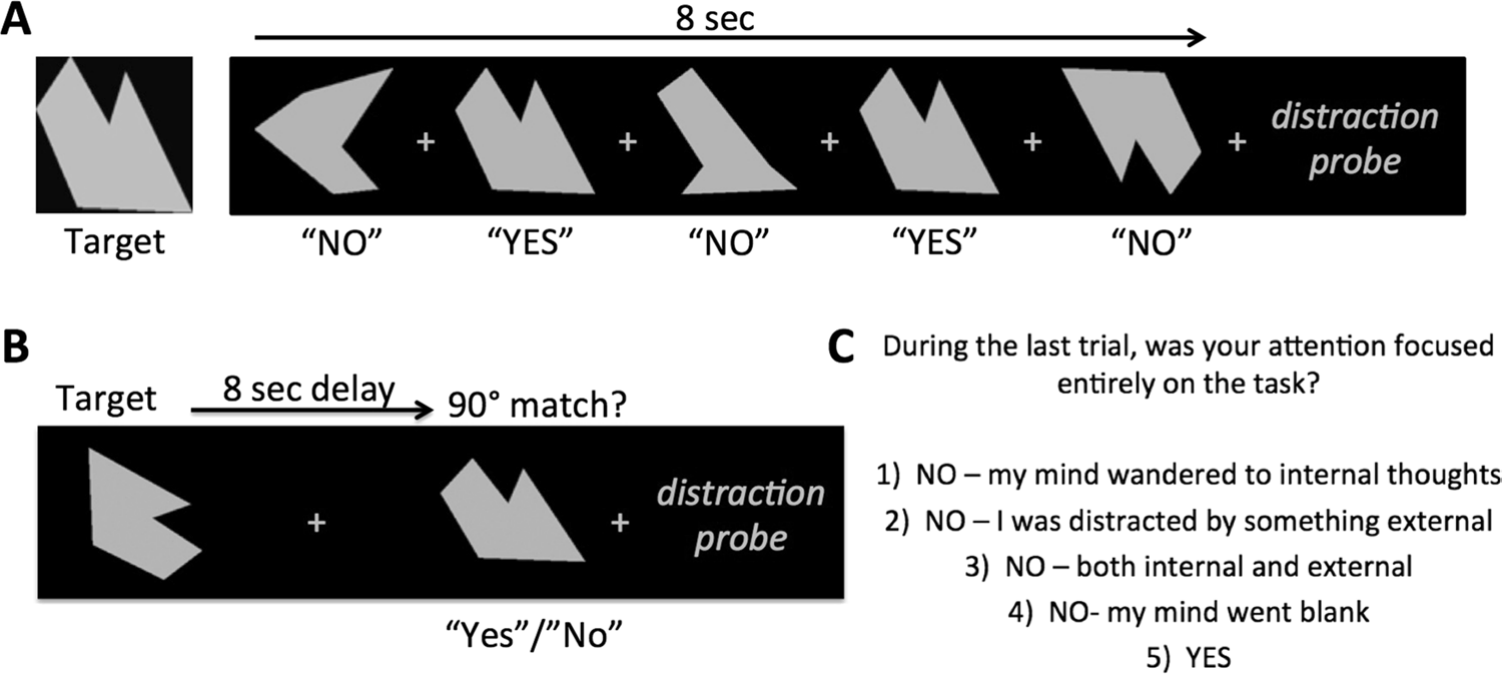
Experimental tasks used to assess (**A**) externally-oriented attention and (**B**) internally-oriented attention; (**C**) distraction probe presented to participants after every 8-sec trial to assess the presence and nature of any distractions that occurred during the previous trial.

For the experimental paradigm, all participants performed two cognitive tasks: an externally-oriented (EXT) visual target detection task and an internally-oriented (INT) mental rotation task (Fig. 1). In order to attempt to equate the cognitive demands of the two tasks, identical stimuli were used for both tasks and a thresholding run was conducted in each participant for each task prior to the experimental runs. The stimuli consisted of abstract gray shapes presented on a black background. All stimuli were presented using PsychoPy v1.76 software and were displayed at 768 × 1024 pixel resolution on an LCD computer monitor positioned approximately 60 cm away from the participant. Auditory stimuli were used in a previous study of auditory distraction on long-term memory^10^, and consisted of 30 segments of recordings made at several public locations (i.e., a busy cafe, a children’s playground, a church mass, and an outdoor sporting event). No sound clips contained discernable words, just ambient sounds from the environment where they were recorded. All of the 8 sec sound clips were band-pass filtered at 1 kHz and their average root-mean-square power spectral densities were normalized in Matlab. Auditory stimuli were presented using Sennheiser HD202 noise-cancelling headphones. In an attempt to subjectively equate volume levels of auditory stimuli across participants, the auditory volume was initially set at a specified volume and then titrated to the point where the participants were able to comprehend several discernable words that were played among ambient background sounds during a pre-experiment test. This procedure is one that we have used successfully in the past^10^ to ensure that all stimuli were presented well above threshold.

### Procedure

The experiment consisted of a thresholding run for each task followed by four blocks of both tasks (INT and EXT), with each block consisting of 30 trials. Two blocks of each task were completed with the addition of background auditory sound and two without sound; participants wore the noise-canceling headphones on all blocks, regardless of the presence or absence of auditory sound. The order of tasks was randomized for each participant.

#### Perceptual Thresholding

The purpose of the thresholding runs was to attempt to equate performance across tasks without sound and individuals as well as across groups of younger and older adults. For the EXT thresholding run, each trial began with participants seeing a target object on the screen and a brief reminder of the instructions. Participants were allowed to view the target item for as long as necessary. Then participants were shown probe items that were either: 1) identical matches to the target, 2) target objects that were rotated by a certain number of degrees, or 3) novel objects. Each probe item appeared for 1000 ms. Participants were instructed to respond “yes” only if a probe matched the target identically, including orientation of rotation. For the INT thresholding run, each trial began by seeing a target object that was presented for 1000 ms and participants were told to mentally rotate the object 90 degrees clockwise. After a 2000 ms delay, participants were shown target objects that were either correctly rotated 90 degrees clockwise or that were rotated by some other number of degrees. If the target matched the 90° rotation, participants were told to respond “yes” and “no” if the rotation deviated from 90°. For both conditions, an adaptive staircase algorithm then adjusted the degree of rotation based on performance: following a correct rejection of a rotated target, the degree of rotation was decreased by 1° on the subsequent trial (thus making it closer to the original target and increasing difficulty); following a false positive response to a rotated target, the degree of rotation was increased by 2° on the subsequent trial. These increments were chosen in order to find a perceptual threshold for each participant where they could perform the task at approximately 70–75% correct. Participants completed a total of 100 thresholding trials each of the EXT and INT tasks. Each participant’s thresholding level was defined as the average degree of rotation for the last 10 trials of each condition, resulting in unique thresholds that were used to set the difficultly levels independently for EXT and INT.

#### External Task

Each EXT trial began with a reminder of the target item (1000 ms), followed by a fixation cross (500 ms), and then presentation of five probes (1000 ms each), interleaved with fixation during an ISI (500 ms), resulting in 8 sec trials (Fig. 1A). For each stimulus, participants responded “yes” if the probe was an identical match to the target and “no” otherwise. Probes consisted of object that were: 1) identical to the target (33% of probes; “yes” response), 2) targets that were rotated the number of degrees determined by EXT thresholding (33%; “no” response), or 3) novel abstract shapes (33%; “no” response). Immediately after each sequence of five probes, participants were given a self-paced distraction probe (see description below), followed by a 1000 ms inter-trial interval.

#### Internal Task

Each 8 sec INT trial began with the presentation of one of 30 abstract shapes (2000 ms), followed by a delay (5000 ms) during which only a fixation cross appeared on a black screen (Fig. 1B). During the delay, participants were told to mentally rotate the target object 90° clockwise. Following the delay, a probe item was presented (1000 ms) and participants responded “yes” if the probe was a correctly rotated target (50% of trials) or “no” if the rotation deviated from 90° (50% of trials). For non-match trials, targets were rotated the number of degrees determined by INT thresholding. Following each trial, participants were given a self-paced distraction probe (see description below), followed by a 1000 ms inter-trial interval.

#### Distraction Probes

After each trial in the two tasks above, participants were presented a distraction probe designed to provide a subjective measure of internal and external distractions during each of the above tasks. Our probe was based on well-validated, experience sampling techniques adapted from previous studies of mind-wandering^5,7,37,44,55,56^. Such indices have been shown to correlate with other laboratory measures and real-world assays of mind-wandering^44–46^. In our experiment, after each trial, participants were asked to report via button press whether their attention was focused entirely on the task during the entire trial. If not, participants were asked to indicate if they were distracted by 1) internal thoughts, 2) something external, 3) both internal and external things, or 4) if their “mind just went blank.” The exact language used in the distraction probe prompt is provide in Fig. 1C.

## Results

### INT and EXT Task Performance

**Figure 2:**
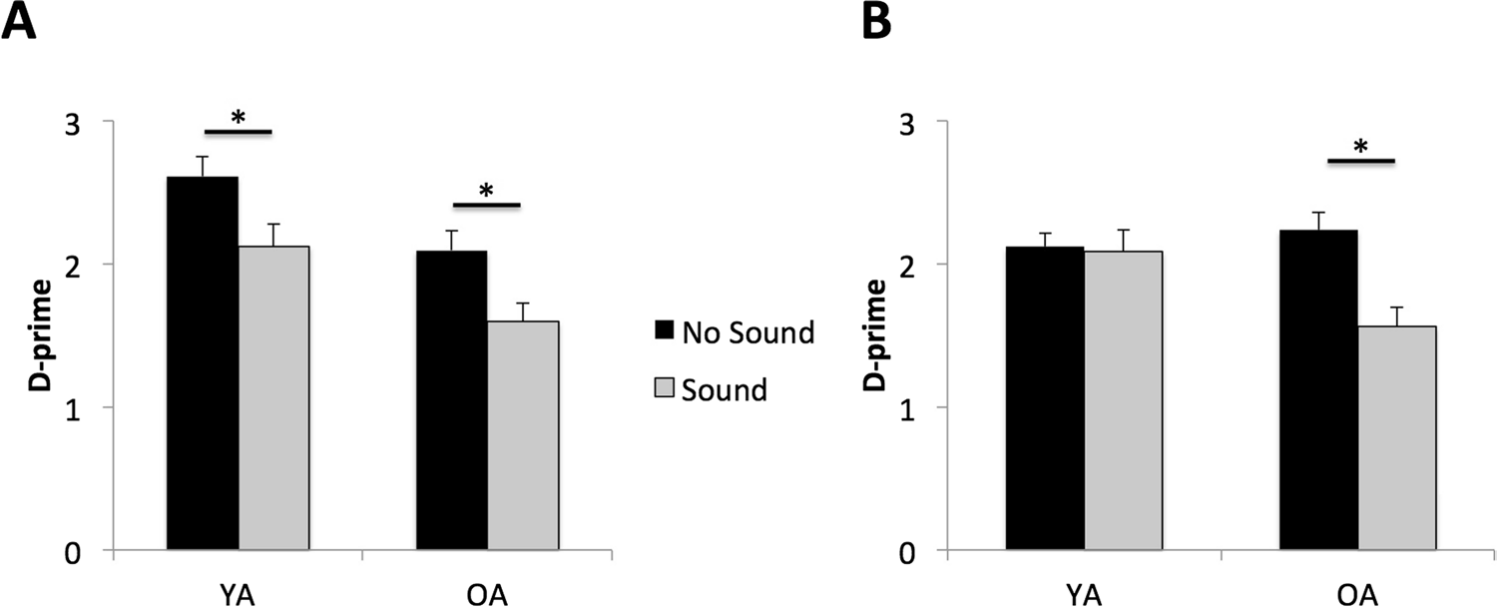
Performance (d′) on the (**A**) external task (EXT), where both groups showed a significant decrease in d′ with auditory sound, and on the (**B**) internal task (INT), where only OA showed a significant decrease in d′ with auditory sound (*p < 0.02; error bars = SEM).

Task performance was indexed using the discrimination metric *d*′^57^, which helped to control for potential differences in false alarms or bias between the two tasks. To assess the impact of age and external auditory sound on task performance between the INT and EXT tasks, we used a 3-way mixed repeated measures multivariate general linear model (GLM) with auditory sound (present or absent) and task orientation (INT or EXT) as the within-subject factors and age group (YA vs OA) as the between subject factor (Fig. 2). In order to help account for minor differences between YA and OA in baseline task performance, we also included each participant’s INT and EXT thresholding level result as continuous covariates. This model revealed a significant 3-way interaction of sound X orientation X age (*F*_*1,43*_ = 14.8, *p* = 0.0004, partial eta^2^ = 0.18). In order to determine whether a particular task orientation was driving the 3-way interaction, additional post-hoc 2-way repeated measures multivariate GLMs were performed separately for *d*′ scores on the EXT and INT. For performance on the EXT task, we did not find a significant age by sound interaction (*F*_*1,45*_ = 0.03, *p* = 0.95, partial eta^2^ = 0.002). We found significant main effects of auditory sound on task performance (*F*_*1,45*_ = 24.0, *p* < 0.001, partial eta^2^ = 0.35), such that both YA and OA showed diminished performance on trials in which the auditory sound was present, and a main effect of age (*F*_*1,45*_ = 9.4, *p* = 0.004, partial eta^2^ = 0.17), such that performance was lower in OA overall, as compared to YA. For YA, *d*′ on the EXT task without auditory sound was 2.6 (*SEM* = 0.14) and with auditory sound it was 2.1 (*SEM* = 0.13). For OA, *d*′ on the task without auditory sound was 2.1 (*SEM* = .15) and with auditory sound it was 1.6 (*SEM* = 0.13). For INT, a significant interaction of age by condition (*F*_*1,45*_ = 9.7, *p* < 0.003, partial eta^2^ = 0.18) revealed that performance in OA was more negatively affected by the presence of auditory sound than was performance in YA (Fig. 2B). Within-subject comparisons confirmed that for YA *d*′ on INT did not differ between the no-sound (mean = 2.12 ± 0.1) and sound (mean = 2.08 ± 0.12) conditions (*t*_23_ = 0.24, *p* = 0.81); whereas OA showed a significant decrease in *d*′ on the sound (mean = 2.24 ± 0.15) as compared to the no-sound (mean = 1.56 ± 0.13) conditions (*t*_22_ = 4.4, *p* = 0.0002).

### Impact of Auditory Sound on Distraction Reports

**Figure 3:**
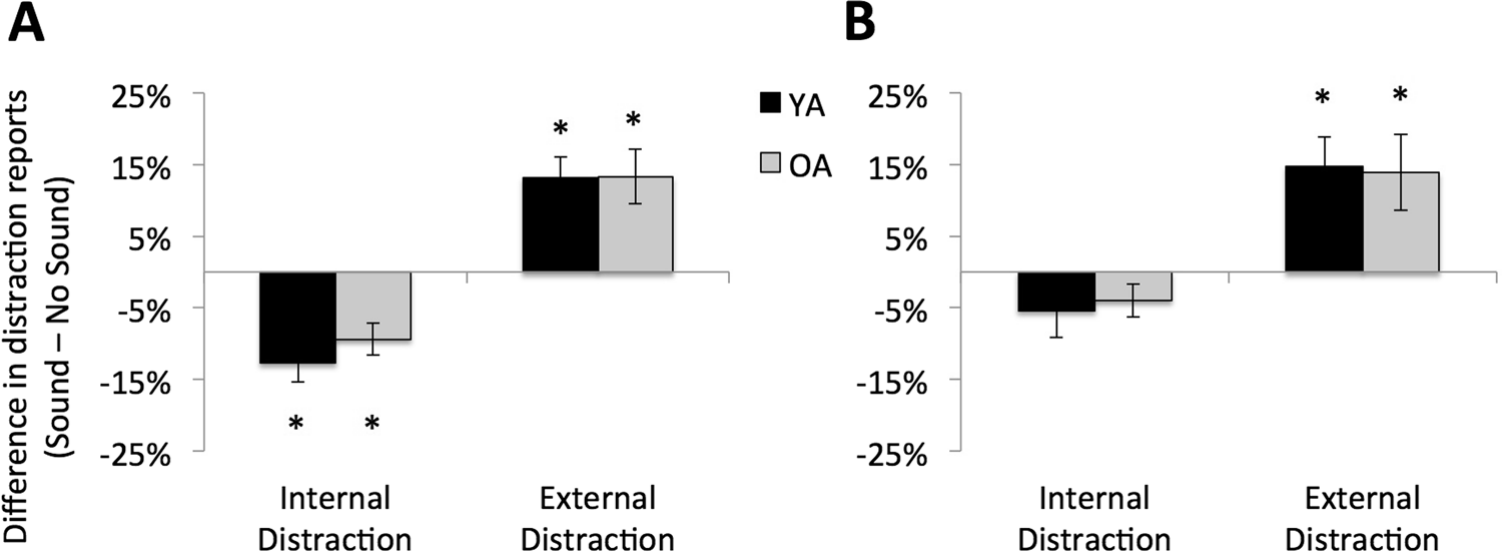
Effect of auditory sound on distraction reports for the (**A**) external task and (**B**) internal task (*one-sample t-test, pcorr < 0.02; error bars = SEM).

With respect to the participant’s distraction reports, our main question of interest was the impact of auditory sound on the relative frequency of self-reported internal and external distractions. We defined internal distractions as a response of “internal” or “both” to the distraction probe and external distraction as responses of “external” or “both”; the frequency of “zone-out” trials was quite low overall (less that 5% of responses in all participants), so this category of distraction was omitted from analysis. Raw frequencies of distraction reports for each category are presented in Table 1. To streamline analyses tailored to our primary hypothesis of interest, we generated difference scores for internal and external distraction reports by subtracting the percentage of trials with each type of distraction in the sound condition from the percentage in the no-sound condition. As a result, we used one-sample *t*-tests for a difference from zero to assess a significant difference in distraction reports between conditions. Due to the number of comparisons, we report both raw and Bonferroni corrected (indicated by “*p*_*corr*_”) statistics. On the EXT task, the presence of external auditory sound led to a decrease in the number of internal distractions and an increase in the number of external distractions in both groups (Fig. 3A). Specifically, in YA the presence of auditory sound led to a 12.6% (*SEM* = 3.0%; *t*_*22*_ = −4.7, *p* = 0.0001, *p*_*corr*_ = 0.0008) reduction of internal distractions and a 13.2% (*SEM* = 2.9%; *t*_*22*_ = 4.4, *p* = 0.0002, *p*_*corr*_ = 0.002) increase of external distractions; in OA, the presence of auditory sound led to a 9% (*SEM* = 2.2%; *t*_*22*_ = −4.3, *p* = 0.0003, *p*_*corr*_ = 0.002) reduction of internal distractions and a 13.3% (*SEM* = 3.8%; *t*_*22*_ = 3.5, *p* = 0.002, *p*_*corr*_ = 0.016) increase of external distractions. On the INT task, the presence of external auditory sound led to an increase in the number of external distractions in both groups (Fig. 3B), but neither group showed a significant decrease in internal distractions. In YA, the presence of auditory sound led to a 5% (*SEM* = 3.3%; *t*_*22*_ = −1.6, *p* = 0.12, *p*_*corr*_ = 0.96) reduction of internal distractions and a 13% (*SEM* = 2.9%; *t*_*22*_ = 4.4, *p* = 0.0002, *p*_*corr*_ = 0.002) increase of external distractions; in OA, the presence of auditory sound led to a 4% (*SEM* = 2.3%; *t*_*22*_ = −2.0, *p* = 0.06, *p*_*corr*_ = 0.48) increase in internal distractions and a 14% (*SEM* = 5.3%; *t*_*22*_ = 3.5, *p* = 0.003, *p*_*corr*_ = 0.024) increase of external distractions.

**Table 1:** Distraction Frequencies.

	Distraction:	Young Adults		Older Adults	
		Internal	External	Internal	External
EXT	No Sound	14	2	11	1
	Sound	6	10	6	9
INT	No Sound	11	1	8	1
	Sound	8	9	3	10

Mean number of internal vs external distractions reported by young and older adults in response to the distraction probes (60 probes total per condition) for the externally-oriented (EXT) and internally-oriented (INT) task performed with or without presentation of auditory sounds.

### Task Performance and Suppression of Internal Distractions

**Figure 4:**
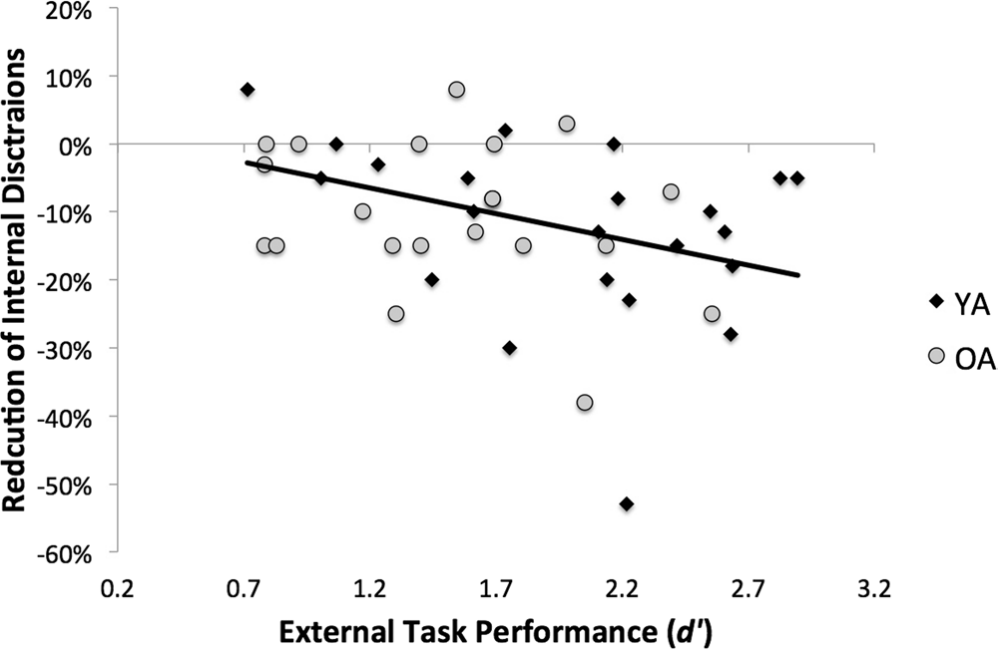
Task Performance and Suppression of Internal Distractions. Reduction of internal distractions between the sound and no-sound conditions predicted task performance on the external task in the presence of auditory sound for both YA (black diamonds) and OA (gray circles).

To determine the extent to which susceptibility to internal distractions impacted task performance, we correlated the difference scores for reports of internal distractions on the sound vs. no-sound conditions with performance on the task conditions that contained external auditory sounds. We did not find a significant correlation between performance on the INT task and change scores for either internal (*r*_*46*_ = 0.14, *p* = 0.35; *p*_*corr*_ = 1.4) or external (*r*_*46*_ = −0.24, *p* = 0.11; *p*_*corr*_ = 0.44) distractions. For EXT, however, we found a significant negative correlation between the percent reduction of internal distractions between sound and no-sound conditions and task performance (*r* = −0.41, *p* = 0.005, *p*_*corr*_ = 0.02), such that in greater suppression of internal distractions in the presence of auditory sound was associated with better task performance (Fig. 4). We did not find a significant correlation between change in external distractions and task performance on EXT (*r*_*46*_ = 0.21, *p* = 0.16; *p*_*corr*_ = 0.64).

## Discussion

In the present study, we sought to determine whether external interference had a differential impact on task performance depending on whether attention was directed internally or externally, and we examined the extent to which internal distractibility, or mind-wandering was affected by the presence of external distractions. We were also interested in understanding how modulation of internal distractibility in the presence of external distraction relates to task performance, and whether these dynamics are affected by healthy aging. Healthy YA and OA performed two tasks—one that was externally-oriented and one with an internally-oriented focus of attention—with and without auditory sound. In terms of performance on the two tasks, we found that auditory sound induced a significant decrease in performance in both young and older adults on the EXT task, and this was accompanied by a shift in the type of distractions reported such that the presence of external auditory sound led to a suppression of internal distractions and increased external distraction. We also found that the degree of suppression of internal distractions predicted successful performance on the EXT task. On the internal task, sound only affected performance in OA and performance did not correlate with changes in internal distraction reports.

### Impact of external sound on task performance and distractibility

Internal and external distractions each can lead to performance decrements on a variety of tasks^15,18,23,30–36,58,59^ but few studies have examined both types of distractions in a single experiment^60,61^. Those studies that have done so report a fairly even split in the frequency of these two types of distractions^62^. Subsequent research has led to the emergence of two distinct views of internal versus external distractions. On the one hand, some researchers have proposed that internal distractions (e.g., mind-wandering) are distinguishable from external distractions both neurally and behaviorally^60,62,63^. Often referred to as the decoupling hypothesis, this line of evidence suggests that the process of being internally distracted is fundamentally different from the process of being externally distracted, and the process of turning one’s attention inward may actually decrease ones propensity to be distracted by external stimuli, by decoupling or disengaging with the external environment. More recently, Unsworth and McMillan (2014) used latent variable analysis to show that, while highly correlated, external and internal distractions (in the form of task-unrelated thoughts) are in fact distinct entities, but also found evidence that when a person is in a state of mind-wandering, they are less likely to be distracted by external stimuli.

While a few studies have sought to characterize the relationship between internal and external distractions, no other studies have, to our knowledge, sampled the frequency of these two types of distractions while experimentally manipulating the amount of external sound present during tasks with differing loci of attentional focus. Rather, most studies of mind-wandering have relied on naturally occurring background sounds as sources of external distraction. In the present study, we manipulated the auditory environment that participants experienced during task engagement, such that it was either quiet or there was auditory sound presented via headphones. In doing so, our study revealed a trade off, or push-pull, relationship between internal and external distractions in both YA & OA in the setting of sound presented during a task. However, this effect was most pronounced when the task had an external orientation (visual target discrimination), where there was an increase in external distractions and a suppression of internal distractions in the presence of external auditory sound. When the orientation of the task was internal, we still saw an increase in the frequency of external distractions in the presence of auditory sound, but without an accompanying suppression of internal distractions. Thus, while much prior research has focused on how the act of focusing ones attention internally can impact susceptibility to external distractions^60,62,63^, here we add to this literature by showing that, when the external sound is added, attention can be pulled significantly away from internal distractions as it is shifted to the external source of distractions. An interesting question is whether there is a critical auditory threshold at which point the background sound begins to have an impact on internal distractibility. The use of a single, standardized, decibel level in the present study precludes further insight into such questions.

Critically, we found that the suppression of internal distractions predicted performance on the externally-oriented task, such that participants who reduced their internal distractions most in the sound condition had the highest task performance. Indeed, we found no clear link between an increase in external distractions and task performance; rather, suppression of internal distractions under the more distracting sound condition appears to have a more prominent association with performance. This finding could provide a possible explanation for why some people actually seek out noisy environments (e.g., coffee shops) to engage in attention-demanding task (e.g., studying or writing). Namely, the presence of external distractions could aid in the suppression of internal distractions, in turn facilitating task performance. Our results would also predict that such behavior would have a differential benefit, depending on the task demands, and performance under externally noisy conditions would likely be associated with improved performance on tasks with an external focus of attention (e.g., using a laptop or mobile device or reading a book) more than tasks with an internal focus (e.g., introspection or mental planning). At the same time, not all individuals feel they benefit from studying in a noisy environment. Unfortunately the present sample size doesn’t allow a thorough examination of the degree to which individual differences might factor into our findings.

Interestingly, internal distractions have been linked to increased activity in the default mode network (DMN)^5,8,62,64^, and activity in this network tends to be negatively correlated with activation of attention networks and with performance on attention-demanding tasks^41,65–67^. A future investigation of brain activity during a cognitive paradigm similar to that employed here would shed light on the nature of the neural underpinnings of internal and external distractions.

### Impact of age on susceptibility to internal and external distractions

Studies in our lab have consistently shown that OA experience deficits in the suppression of externally-presented distracting information^31^, that these deficits occur at early visual processing stages^18,32,36,52^ and that this is mediated by a failure to maintain functional connectivity between prefrontal and visual cortices^31^. This susceptibility to external distractions in OA leads to disruption of performance in numerous domains, including working memory^18,32^, attention^68^, and long-term memory^10,11^. In contrast to this deficit in external distraction suppression, OA have been shown to exhibit fewer internal distractions during certain tasks compared to young adults^49,50,69,70^. This potential discrepancy between deficits in internal and external distractor suppression in OA clearly warrants exploration. Others have proposed that the apparent decrease in mind wandering frequency in OA actually stems from diminished cognitive control^51^.

Indeed, there is evidence that ‘mind-wandering’ reflects both state-dependent changes in cognitive status, varying inversely with both task difficulty and arousal^37,55^, and trait-level individual differences in executive function^44^. In general, the frequency of reporting internal distractions is inversely correlated with executive processes. Mind-wandering increases as tasks become well-practiced^58^; it does not affect performance on easy, mundane tasks, but negatively impacts tasks that require cognitive control^38^. Further, the frequency of internal distractions correlates negatively with WM capacity^7,8,44^. A growing body of research implicates inefficient regulation of cognitive control processes as a core mechanism underlying many of the cognitive deficits associated with healthy aging^47,71–75^. Taken together, these findings have led to the notion that OA may have insufficient cognitive resources to maintain both task-relevant and irrelevant thoughts during task performance. Such a view would be consistent with the idea that mind-wandering results from cognitive control failures regardless of age. According to the reduced cognitive resources theory of aging, OA may have fewer overall cognitive resources than YA. When engaged in an attention-demanding task, OA thus invest more of their available resources into performing the task, and have less ability to simultaneously engage in mind-wandering or internal thoughts that are not task-relevant^59^.

Our findings add to this literature on the nature of internal distractions in healthy aging. On the one hand, we found no difference in the impact of external sound on the relative frequencies of internal versus external distraction reports between YA and OA. Both groups showed a shift from more internal distractions under relatively quiet task conditions to increased external distractions (and fewer internal distractions) when faced with externally-presented auditory sound. Interestingly, on the EXT task, both groups exhibited a performance cost associated with the auditory sound and external distractions, whereas on the internally-oriented task, only OA showed a cost related to external sound. This finding raises the intriguing possibility that there may be differential effects of age on the interaction between distraction and the ability to perform attentionally-demanding tasks depending on the focus of the task at hand.

To our knowledge, no other studies have examined the impact of age on two separate tasks with differing (internal vs external) orientations, with and without the presence of auditory distractions. From this parametric examination, we were able to determine that OA appear to be more susceptible to distractions, regardless of task orientation, whereas YA seem to be more resilient to distractions on a task with an internal orientation. Previous research in our lab has shown that when OA are faced with a need to multitask, they tend to be more impaired on a concurrent working memory task (i.e., an internally-oriented task), when compared to YA^76^. Further, while YA are quickly able to reengage functional connectivity between the networks associated with the working memory trace, OA appear less able to reengage and the network remains uncoupled following distraction. The present findings may shed new light on the question of whether there are separable, even dissociable, neurocognitive systems underlying internal and external attention^2,3^. A possible explanation for our current findings is that performing a task with an internal orientation while listening to distracting background noise constitutes a form of task-switching, and, consistent with previous reports, OA are more impaired on such tasks^16,76,77^. In contrast, performing an externally oriented task may require less switching between systems, but may place an undue burden on the limited resources available for processing external stimuli, thus leading to decrements in performance in both YA and OA. Future studies with neuroimaging measures would help to clarify the nature of the neural systems that are at play during performance of tasks with internal vs external orientations.

It should be noted that the present study has some limitations. The sample size was relatively small and, while many of the magnitudes of behavioral effects were large, others were only modest, and thus warrant additional exploration and replication in future studies employing larger samples. In addition, it should be noted that our participants did not undergo formal screening for hearing acuity/impairment. While we did attempt to subjectively equate volume levels of auditory stimuli across participants, ensuring that all stimuli were presented well above threshold, we cannot rule out the possibility that differences in hearing might have impacted our results. Future studies of auditory distraction in older adults should include a more formal test of hearing abilities.
